# ‘Scarless’ removal of forehead lipomas

**DOI:** 10.1308/003588412X13171221591259c

**Published:** 2012-05

**Authors:** A Fattah

**Affiliations:** Broomfield Hospital, ChelmsfordUK

## BACKGROUND

Traditionally, excision of forehead lipomas is performed via a transverse incision camouflaged in a forehead crease. This approach necessarily violates the frontalis, under which these lipomas reside.^1^ Additionally, transection of the overlying supraorbital and supratrochlear nerves leads to paraesthesia of the forehead and anterior scalp. To minimise such morbidity, an indirect approach is advocated that can be performed as a day case local anaesthetic procedure.

## TECHNIQUE

**Figure 1:**
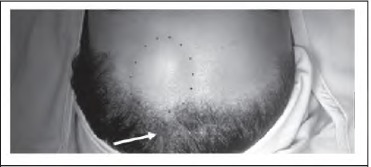
The border of the lipoma is marked (dots) and a vertical incision (arrow) is made behind the hairline.

**Figure 2:**
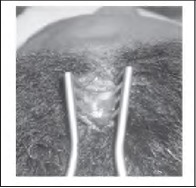
The lipoma has been ‘pushed’ up into the hairline incision by sliding a finger along the forehead.

**Figure 3:**
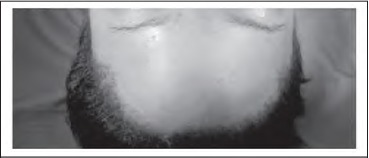
There are no visible forehead scars.

The border of the lipoma is marked (Fig 1; dots) and a subgaleal field block of local anaesthesia is infiltrated from the scalp incision down towards the forehead in the subgaleal plane to achieve hydrodissection. A vertical incision (Fig 1; arrow) is made 1cm behind the hairline through the galea to reach the loose areolar subgaleal (and subfrontal) plane. Blunt dissection deep to the frontalis preserves the supraor-bital and supratrochlear nerve branches and the lipoma is released from the surrounding tissues using blunt ‘spreading scissor’ dissection. Digital pressure (exerted by sliding the finger along the forehead from inferior to superior) allows the lipoma to be ‘pushed’ up into the hairline incision (Fig 2). A drain is not required as there is minimal bleeding when accessing the lipoma via this plane. Using this technique, visible forehead scars are eliminated (Fig 3).

## DISCUSSION

**Figure 4:**
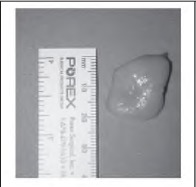
The lipoma is delivered intact.

While similar results can be achieved with liposuction^2^ or endoscopi- cally,^3^ the described method is cheaper, faster and delivers the lipoma intact (Fig 4). This method is suitable for lipomas in the upper two thirds of the forehead; below this, the contour of the forehead presents a difficult approach without additional instrumentation.
